# Cinnamon induces browning in subcutaneous adipocytes

**DOI:** 10.1038/s41598-017-02263-5

**Published:** 2017-05-26

**Authors:** Hiu Yee Kwan, Jiahui Wu, Tao Su, Xiao-Juan Chao, Bin Liu, Xiuqiong Fu, Chi Leung Chan, Rebecca Hiu Ying Lau, Anfernee Kai Wing Tse, Quan Bin Han, Wang Fun Fong, Zhi-ling Yu

**Affiliations:** 1 Centre for Cancer and Inflammation Research, School of Chinese Medicine, Hong Kong Baptist University, Hong Kong, China; 2 Institute of Integrated Bioinfomedicine & Translational Science, HKBU Shenzhen Research Institute and Continuing Education, Shenzhen, China; 3 Guangzhou Institute of Cardiovascular Disease, Guangzhou Key Laboratory of Cardiovascular Disease, and the Second Affiliated Hospital, Guangzhou Medical University, Guangzhou, China

## Abstract

Browning is the process of increasing the number of brite cells, which helps to increase energy expenditure and reduce obesity. Consumption of natural and non-toxic herbal extracts that possess the browning effect is an attractive anti-obesity strategy. In this study, we examined the browning effect of cinnamon extract. We found that cinnamon extract (CE) induced typical brown adipocyte multiocular phenotype in 3T3-L1 adipocytes. The treatment also increased brown adipocytes markers and reduced white adipocytes markers in the 3T3-L1 adipocytes. In *ex vivo* studies, we found that CE increased brown adipocytes markers in the subcutaneous adipocytes isolated from db/db mice and diet-induced obesity (DIO) mice. However, CE did not significantly affect UCP1 expression in the adipocytes isolated from perinephric adipose tissue and epididymal adipose tissue. β3-adernergic receptor (β3-AR) antagonist reduced the CE-enhanced UCP1 expression, suggesting an involvement of the β3-AR activity. Oral administration of CE significantly increased UCP1 expression in the subcutaneous adipose tissue *in vivo* and reduced the body weight of the DIO mice. Taken together, our data suggest that CE has a browning effect in subcutaneous adipocytes. Our study suggests a natural non-toxic herbal remedy to reduce obesity.

## Introduction

Obesity has become a public health crisis. Obese people are predisposed to a number of diseases, including cardiovascular disease, type 2 diabetes, hypertension, stroke and many types of cancers^1^. Strategies that can enhance energy expenditure and combat the epidemic of obesity are desperately needed.

There are two types of adipocytes, white adipocyte and brown adipocyte. White adipocytes are specialized to store chemical energy while brown adipocytes dissipate energy as heat. The thermogenic responses in brown adipocytes are dependent on the abundance of uncoupling protein 1 (UCP1) in the mitochondria^2^. UCP1 is an integral membrane protein unique to mitochondria in brown adipocytes and is responsible for the respiratory uncoupling during thermogenesis in the brown adipocytes. However, active brown fat is virtually absent or has low thermogenesis activity in obese people.

Browning is the induction of brown adipocyte markers including UCP1 in white adipocytes, these brown adipocyte-like cell are called brite cells or beige cells^3, 4^. These brite cells can be found in the white adipose tissues in rodents and human^3, 4^. Cold stress or in response to treatment with β3 selective adrenergic agonist increases the development of these brite cells in the white adipose tissue^5, 6^ although the origin of these brite cells is still controversial^7^. Abundance of brite or beige cells in white adipose tissue shows resistance to diet-induced obesity and improved glucose metabolism^8–11^.

Cinnamon is a spice produced from the bark of trees from the genus *Cinnamomum* (*Cinnamomum zeylanicum* and *Cinnamon cassia*) and belongs to the Lauraceae family^12^. Cinnamon is one of the most important spices used daily by people all over the world. Cinnamon exerts antioxidant, anti-inflammatory, antimicrobial and anticancer effects^12, 13^. Furthermore, cinnamon extract improves insulin sensitivity and has beneficial effects on the metabolism^14, 15^. A recent study suggests that cinnamon extract reduces lipid and glycogen accumulation in the livers of high fat diet-fed animal models and lowers glucose levels by increasing insulin secretion^16–18^. Other study showed that cinnamon extract regulated expression of multiple genes related to carbohydrate metabolism and lipogenesis in the adipose tissue of fructose-fed rats and had anti-hyperglycemic and anti-hyperlipidemic effects in diabetic animal models^19^. The anti-obesity effect of the extract is less studied. A study showed that the extract increased expression of glucose transporter in adipocytes^20^. However, the effects of the cinnamon extract on adipocyte differentiation are inconsistent and controversial^21^.

Our study aimed to find out the browning effect of the cinnamon extract (CE) with *in vitro, ex vivo* and *in vivo* studies. We showed that CE increased expressions of UCP1 and other brown adipocyte markers in subcutaneous adipocytes, β3-AR activity was involved in the CE-induced browning process.

## Results

### Cinnamon extract (CE) induces typical brown adipocyte multiocular phenotype in 3T3-L1 cells

**Figure 1:**
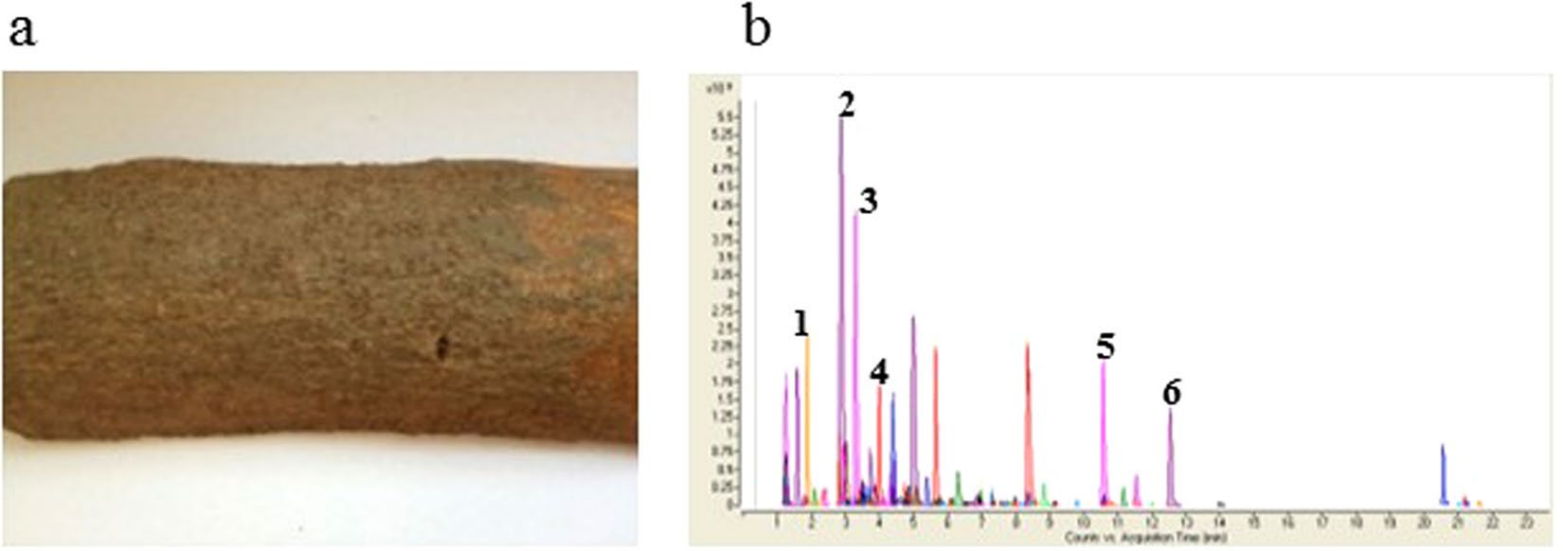
Quality control of cinnamon extract (CE) by HPLC-ESI-MS. (**a**) Cinnamon (bark of Cinnamomum cassia); (**b**) markers for CE: (1) protocatechuic acid, (2) catechin, (3) chlorogenic acid, (4) aesculetin, (5) quercetin and (6) icariin.

Cinnamon, the bark of *Cinnamomum cassia* (Fig. 1a), was extracted by 70% ethanol in water and the dried cinnamon extract (CE) powder was reconstituted in DMSO for experiments. We first performed HPLC-ESI-MS to determine the chemical composition of CE for quality control. We have identified and quantified some of the bioactive compounds in the CE (Fig. 1b) with standards purchased from NICPBP National Institute for the Control of Pharmaceutical and Biological Products, China. The identified compounds in the CE are protocatechuic acid of 17.3033 mg/kg, catechin 302.0128 mg/kg, chlorogenic acid 25.1002 mg/kg, aesculetin 12.7499 mg/kg, quercetin 180.9573 mg/kg and icariin 51.1641 mg/kg.

**Figure 2:**
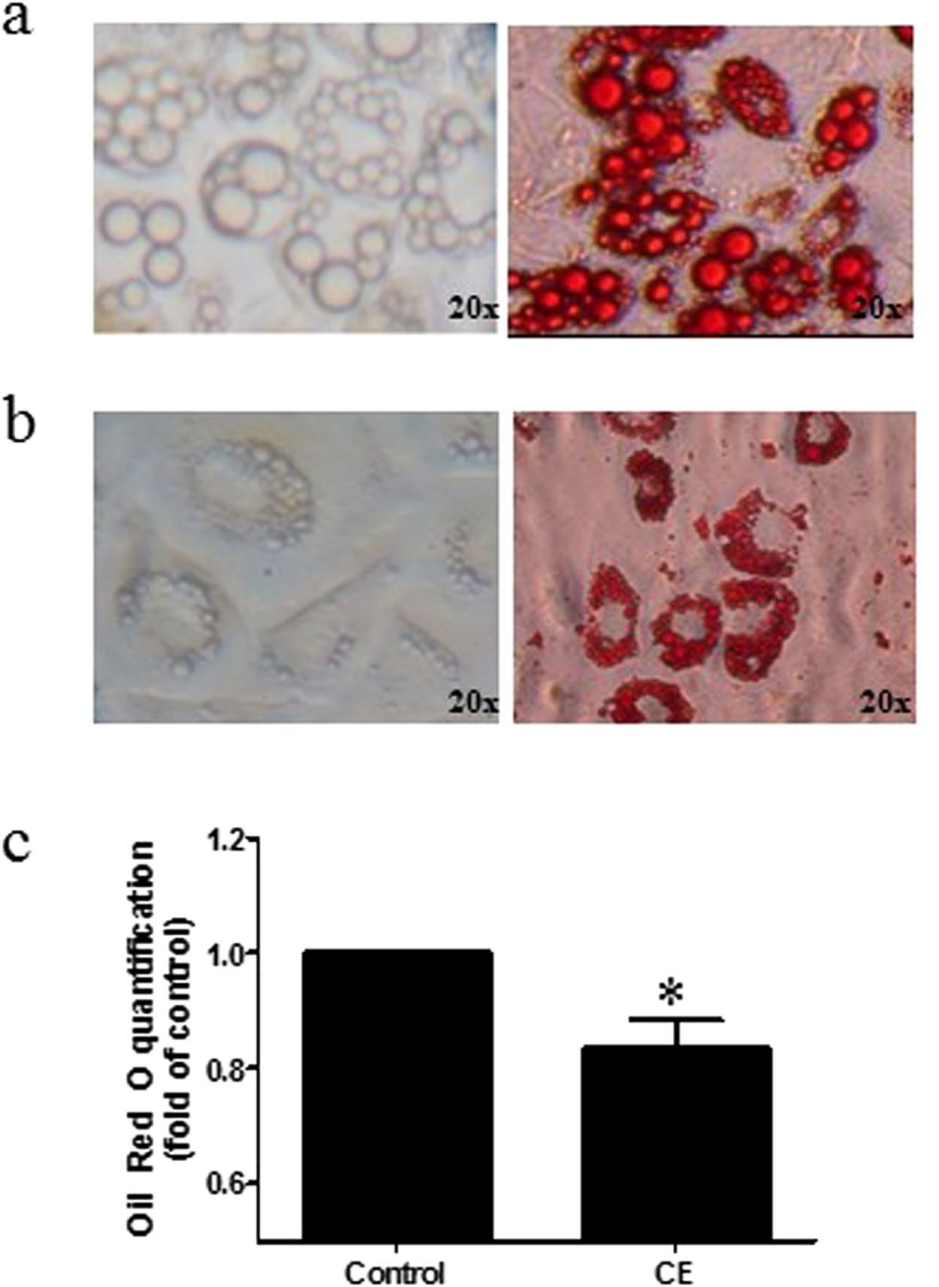
Oil Red O staining of (**a**) 3T3-L1 adipocytes and (**b**) CE-treated 3T3-L1 adipocytes. (**c**) Lipid levels in control and CE-treated 3T3-L1 adipocytes. CE, cinnamon extract (80 µg/ml). Original magnification 20x. *p < 0.05, n = 3 individual experiments.

We differentiated mouse embryo fibroblast 3T3-L1 to mature adipocytes for the study. Figure 2a showed the typical phenotype of white adipocytes, lipid droplets were stained by Oil Red O. We added CE (80 µg/ml) to the 3T3-L1 adipocytes on day 6 during the course of differentiation, typical brown adipocyte multiocular phenotype was observed after 24-hr incubation (Fig. 2b). The lipid content in the 3T3-L1 adipocytes is significantly reduced upon the CE treatment (Fig. 2c).

### CE increases brown adipocyte marker gene expressions in 3T3-L1 adipocytes

**Figure 3:**
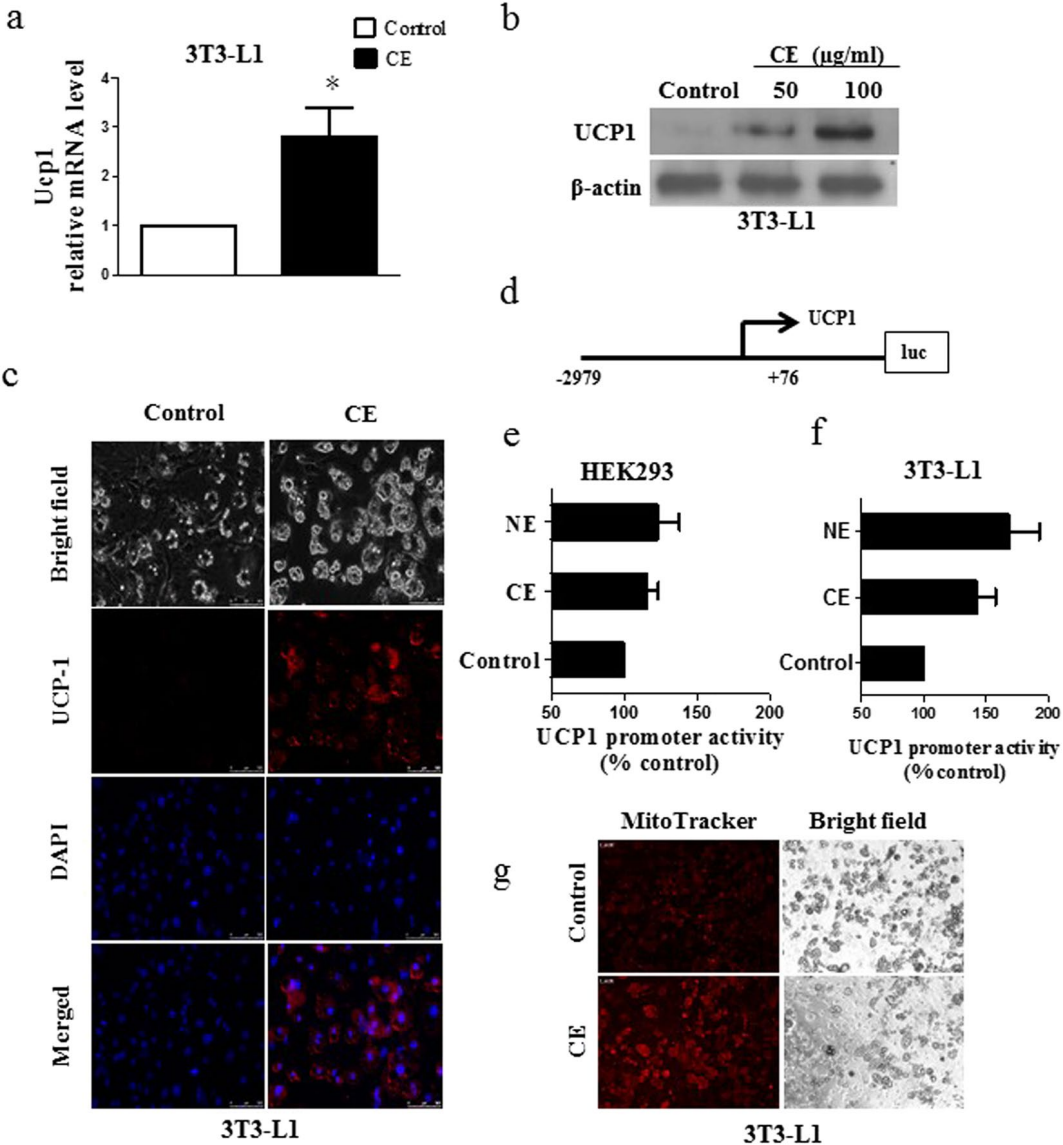
(**a**) Ucp1 mRNA expression, (**b**) UCP1 protein expression and (**c**) immunofluorescence staining of UCP1 in vehicle control and CE-treated 3T3-L1 cells. (**d**) Ucp1 promoter construct (−2979 to base +76) ligated into pGL3-basic luciferase reporter vector. Ucp1 promoter activity in (**e**) HEK293 cells and (**f**) 3T3-L1 cells. (**g**) MitoTracker Green (Molecular Probe) staining of the mitochondria in control and CE-treated 3T3-L1 cells. CE, cinnamon extract (80 µg/ml); NE, norepinephrine (10 µM). Ucp1, uncoupling protein 1; DAPI, (4′,6-diamidino-2-phenylindole staining). *p < 0.05, n = 3 individual experiments. Original magnification 20x.

**Figure 4:**
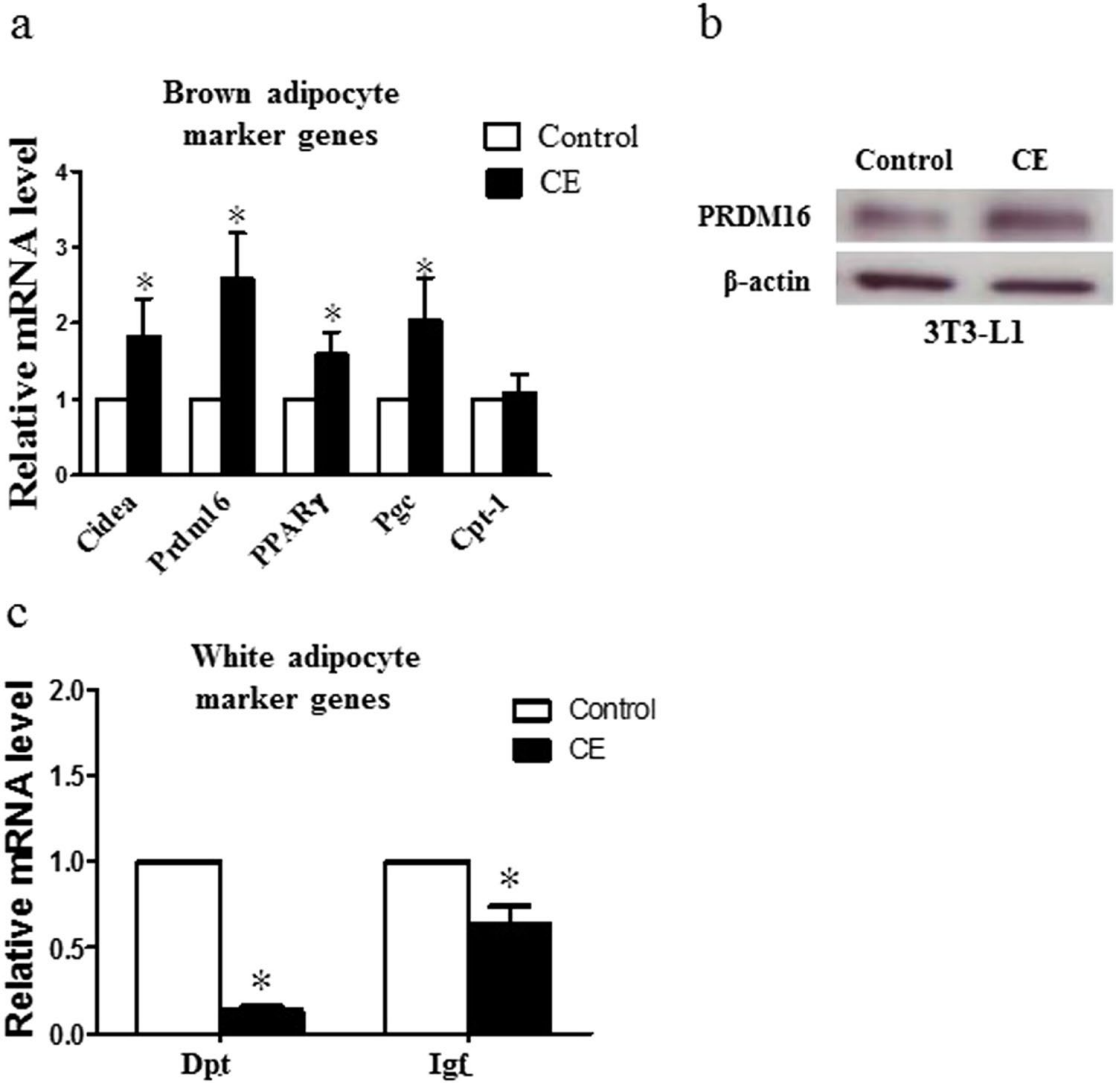
Expressions of (**a**) brown adipocyte marker genes and (**c**) white adipocyte marker genes in control and CE-treated 3T3-L1 cells. (**b**) Protein expression of PRDM16 in control and CE-treated 3T3-L1 adipocytes. CE, cinnamon extract (80 µg/ml). Cidea, cell death-inducing DFFA-like effector A; Prdm16, PR domain containing 16; PPARγ, peroxisome proliferator-activated receptor gamma; Pgc, PPARγ coactivator-1; Cpt-1, carnitine palmitoyltransferase; Dpt, dermatopontin; Igf, insulin-like growth factor. *p < 0.05, n = 3 individual experiments.

We also found that CE significantly increased Ucp1 mRNA levels (Fig. 3a) and protein levels (Fig. 3b and Fig. S1) in 3T3-L1 adipocytes; the increase of UCP1 expression upon CE treatment is also demonstrated by immunofluorescence staining (Fig. 3c). Since CE increased Ucp1 mRNA level, we next examined if CE affected Ucp1 promoter activity. We transfected a Ucp1 −2979 bp reporter plasmid (Fig. 3d) to HEK293 cells and 3T3-L1 adipocytes, respectively. We found that the CE increased UCP1 promoter activities in these two cell models (Fig. 3e and f). Norepinephrine (NE)^22^ was used as a positive control to increase the UCP1 promoter activity in these cells (Fig. 3e and f). Since UCP1 is an integral membrane protein in the mitochondria, we used MitoTracker Green (Molecular Probes, Invitrogen USA) to stain mitochondria in the 3T3-L1 adipocytes to examine if CE enhanced mitochondrial biogenesis. As shown in Fig. 3g, CE increased mitochondrial protein biogenesis as indicated by a stronger staining in the CE-treated 3T3-L1 adipocytes when compared to the vehicle control adipocytes. Furthermore, CE also increased the expressions of other brown adipocyte marker genes including cell death-inducing DFFA-like effector A (Cidea), PR domain containing 16 (Prdm16), peroxisome proliferator-activated receptor gamma (PPARγ), PPARγ coactivator-1 (Pgc) and the fatty acid oxidation marker gene carnitine palmitoyltransferase 1 (Cpt1) (Fig. 4a). The enhanced PRDM16 is further suggested by its elevated protein level in the CE-treated 3T3-L1 adipocytes (Fig. 4b and Fig. S2). CE also reduced white adipocyte marker genes including dermatopontin (Dpt) and insulin-like growth factor (Igf) (Fig. 4c) in the 3T3-L1 adipocytes. All these data suggest that CE induces browning in 3T3-L1 adipocytes.

### CE increases brown adipocyte maker gene expressions in subcutaneous adipocytes isolated from C57BLKS db/db mice

**Figure 5:**
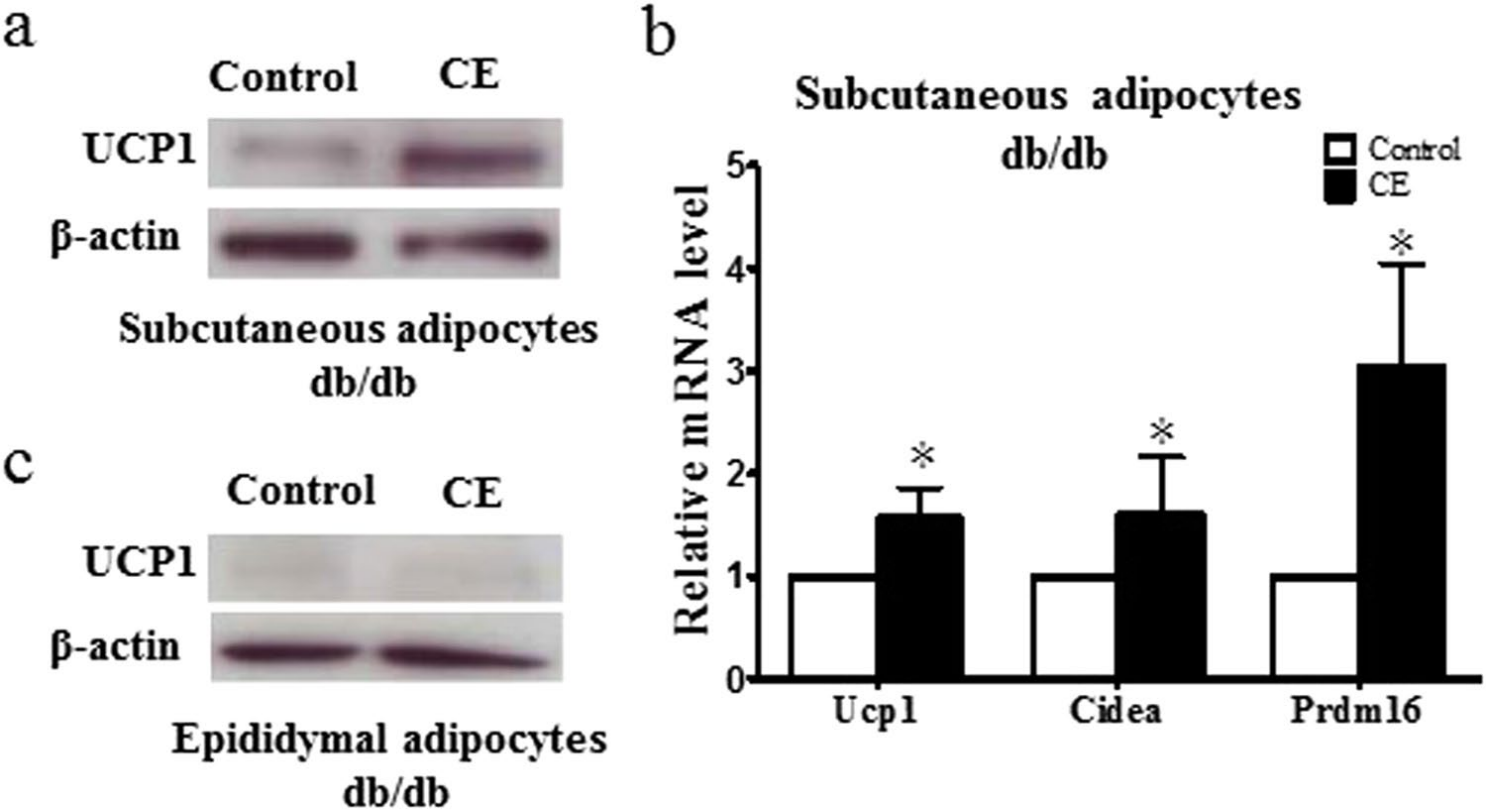
Expressions of (**a**) UCP1 protein and (**b**) brown adipocyte marker genes in subcutaneous adipocytes isolated from db/db mice. (**c**) Expression of UCP1 protein in epididymal adipocytes isolated from db/db mice. CE, cinnamon extract (80 µg/ml). Ucp1, uncoupling protein 1; cidea, cell death-inducing DFFA-like effector A; Prdm16, PR domain containing 16. *p < 0.05, n = 3 individual experiments.

To study the browning effect of CE *ex vivo*, we dissected subcutaneous adipose tissues from db/db mice. However, adipose tissue contains many cell types, including adipocytes, endothelial cells, preadipocytes and fibroblasts, it is difficult to determine whether the effect of CE is a direct action on adipocytes or an indirect action mediated by other cell types. Therefore, we freshly isolated mature adipocytes from subcutaneous adipose tissue dissected from db/db mice. We then treated these freshly isolated adipocytes with CE for 24 hr. We found that CE increased UCP1 protein (Fig. 5a and Fig. S3) and mRNA (Fig. 5b) expressions in these subcutaneous adipocytes, and other brown adipocyte markers including Cidea and Prdm16 (Fig. 5b). However, the CE treatment did not increase UCP1 expression in epididymal adipocytes isolated from these mice (Fig. 5c and Fig. S4). These results suggest that CE induces browning in subcutaneous adipocytes in db/db mice.

### CE increases brown adipocyte marker gene expressions in subcutaneous adipocytes isolated from DIO mice

**Figure 6:**
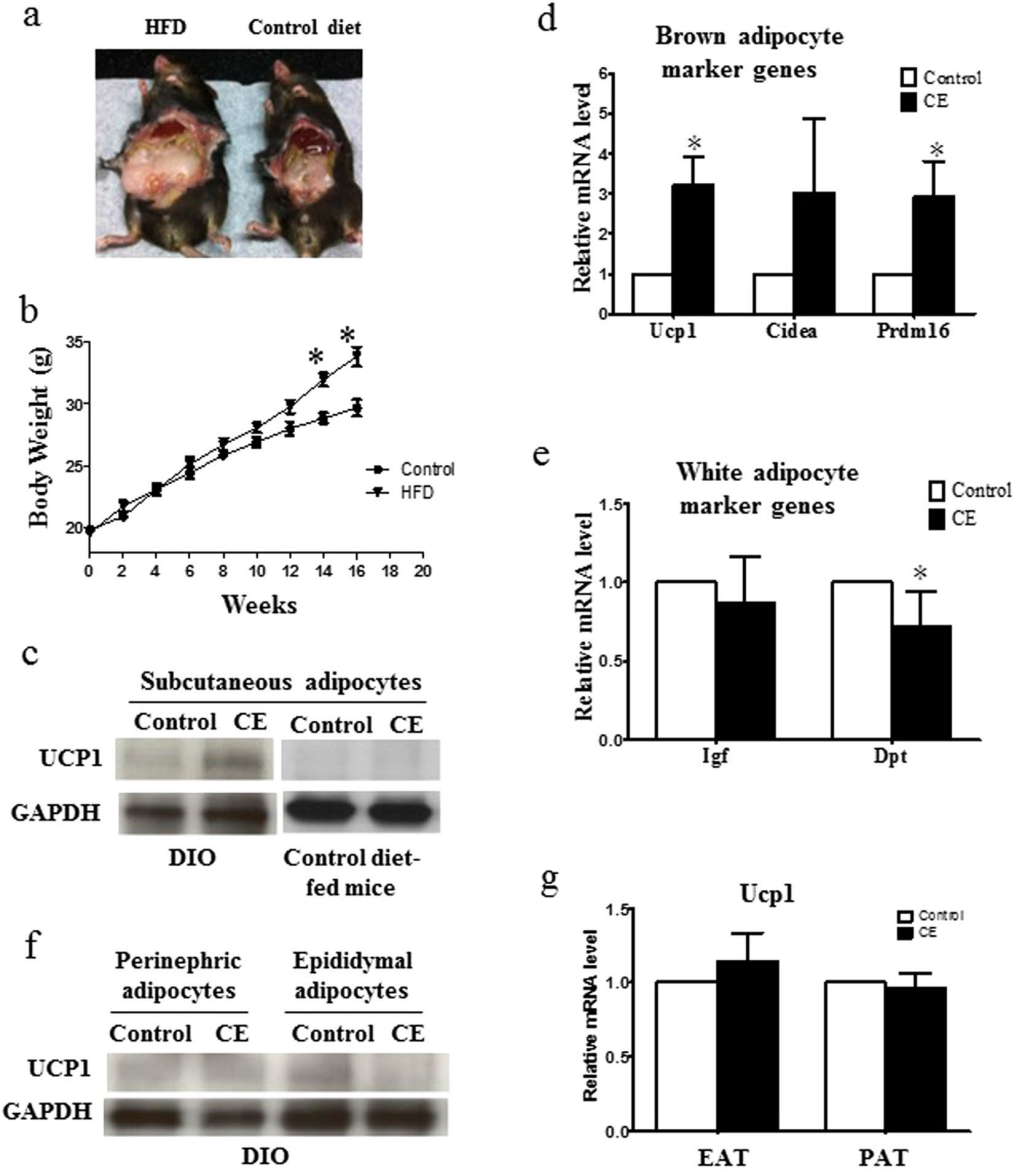
(**a**) High fat diet (HFD)-induced obesity mouse (DIO) and matched control diet-fed mouse. (**b**) Body weights of DIO mice and control diet-fed mice. (**c**) UCP1 protein expression in subcutaneous adipocytes isolated from the DIO mice and control diet-fed mice. (**d**) Expressions of brown adipocyte marker genes and (**e**) white adipocyte marker genes in subcutaneous adipocytes isolated from the DIO mice. (**f**) Protein expression and (**g**) mRNA expression of UCP1 in perinephric adipocytes (PAT) and epididymal adipocytes (EAT) isolated from the DIO mice. CE, cinnamon extract (80 µg/ml). Ucp1, uncoupling protein 1; Cidea, cell death-inducing DFFA-like effector A; Prdm16, PR domain containing 16. *p < 0.05, n = 3 individual experiments.

Transgenic mice such as db/db mice might have developmental compensation and non-specific effects. In contrast, diet-induced obesity (DIO) mice might have similar physiology to human obesity and truly reflect the responsiveness to CE treatment. Therefore, we included DIO mice as another obesity model in the study. We fed 5-week-old C57BL/6 mice with high fat diet to induce DIO mice while control mice were fed matched control diet (Fig. 6a). Starting from the 14^th^ week of the dietary intervention, the body weight of the DIO mice was significantly greater than that of the matched control-diet-fed mice (Fig. 6b). We then isolated the subcutaneous adipocytes from these mice and treated these adipocytes with CE. We found that CE increased UCP1 expression in the subcutaneous adipocytes isolated from DIO mice (Fig. 6c). The treatment also increased expressions of other brown adipocyte marker genes (Fig. 6d) and decreased expressions of white adipocyte marker genes (Fig. 6e). However, CE did not significantly increase the UCP1 expression in the subcutaneous adipocytes isolated from the matched control-diet-fed mice (Fig. 6c and Fig. S5). Since the propensity to express UCP1 upon stimulation differs between various white adipose tissue depots^23^; we examined if CE increased UCP1 expression in adipocytes isolated from other fat depots. Interestingly, we found that CE treatment neither increase UCP1 protein level (Fig. 6f and Fig. S6) nor the mRNA level (Fig. 6g) in adipocytes isolated from perinephric adipose tissue and epididymal adipose tissue in these DIO mice. These results suggest that CE has a browning effect in the subcutaneous adipocytes in DIO mice.

### CE increases UCP1 expression by activating β3-adrenergic receptor

**Figure 7:**
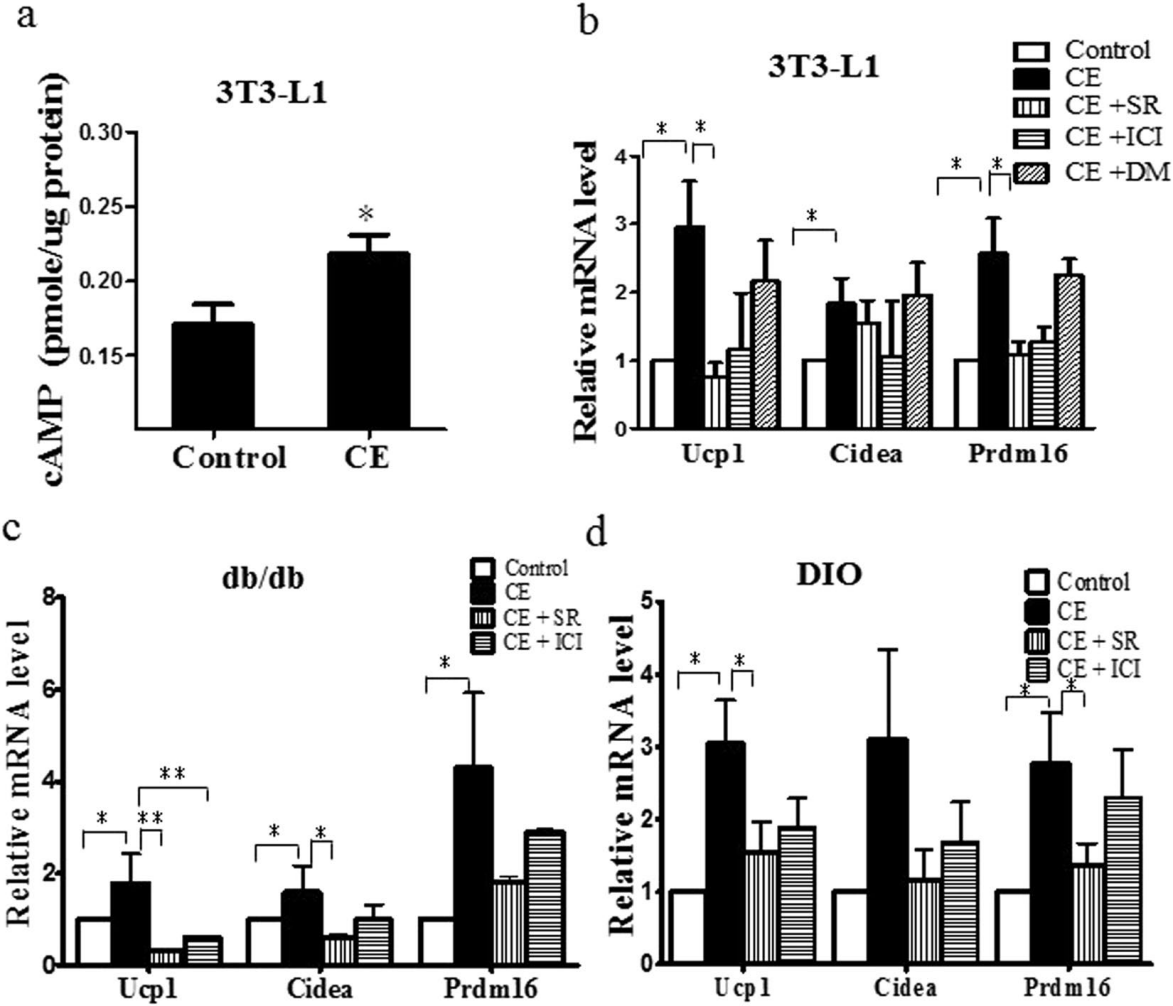
(**a**) cyclic AMP (cAMP) levels in control and CE-treated 3T3-L1 cells. Expressions of brown adipocyte marker genes in (**b**) 3T3-L1 cells, (**c**) subcutaneous adipocytes isolated from db/db mice and (**d**) subcutaneous adipocytes isolated from diet induced obesity (DIO) mice. CE, cinnamon extract (80 µg/ml); SR, SR59230A (1 µM); ICI, ICI118551 (1 mM); doxazosin mesylate, DM (1 µM). Ucp1, uncoupling protein 1; Cidea, cell death-inducing DFFA-like effector A; Prdm16, PR domain containing 16. *p < 0.05, **p < 0.01, n = 3 individual experiments.

In adipocytes, UCP1 expression can be increased by β-adrenergic stimulation *via* the cyclic AMP (cAMP)-mediated pathways^5, 24^. Therefore, we examined if CE-increased UCP1 expression was mediated by the β-adrenergic signaling. We treated the 3T3-L1 adipocytes with CE and found that cAMP levels in these adipocytes were increased (Fig. 7a), implying an involvement of the β-adrenergic receptor (β-AR) activity. Next, we preincubated the 3T3-L1 adipocytes with either β2-AR antagonist ICI1185551 (Sigma-Aldrich) or β3-AR antagonist SR59230A (Sigma-Aldrich), before we treated the adipocytes with CE. ICI 118,551 possesses a high degree of selectivity and specificity for the beta 2-adrenoceptor^25^. We found that inhibition of β2-AR did not have significant effect on the Ucp1 expression (Fig. 7b). However, inhibition of β3-AR significantly reduced the CE-enhanced Ucp1 and Prdm16 expression in the 3T3-L1 adipocytes (Fig. 7b). SR 59230 A is a selective antagonist of the β3-AR, but is subsequently shown to also act at α1-AR at high doses. To ensure the involvement of β3 but not α1 in the CE-enhanced UCP1 expression, we used doxazosin mesylate (Tocris) which is a selective α1-AR. We found that inhibition of α1-AR did not have significant effect on Ucp1 expression (Fig. 7b). Taken together, our data suggest that β3-adrenergic receptor plays a role in the CE-enhanced Ucp1 expression in 3T3-L1 adipocytes.

To further suggest the involvement of β3-AR in the CE-enhanced Ucp1 expression in white adipocytes, we isolated the adipocytes from the subcutaneous adipose tissue dissected from db/db mice and from DIO mice, respectively. Then, we pre-treated these adipocytes with either β2-AR antagonist ICI118551 or β3-AR antagonist SR59230A, before we treated them with CE. We found that inhibition of β3-AR significantly reduced the CE-enhanced Ucp1 expression in the subcutaneous adipocytes dissected from db/db mice (Fig. 7c) and from DIO mice (Fig. 7d). These results clearly demonstrated that β3-AR was involved in the CE-enhanced Ucp1 expression in the subcutaneous adipocytes.

### Oral administration of CE reduces body weight and increases UCP1 expression in the subcutaneous adipocytes in DIO mice

**Figure 8:**
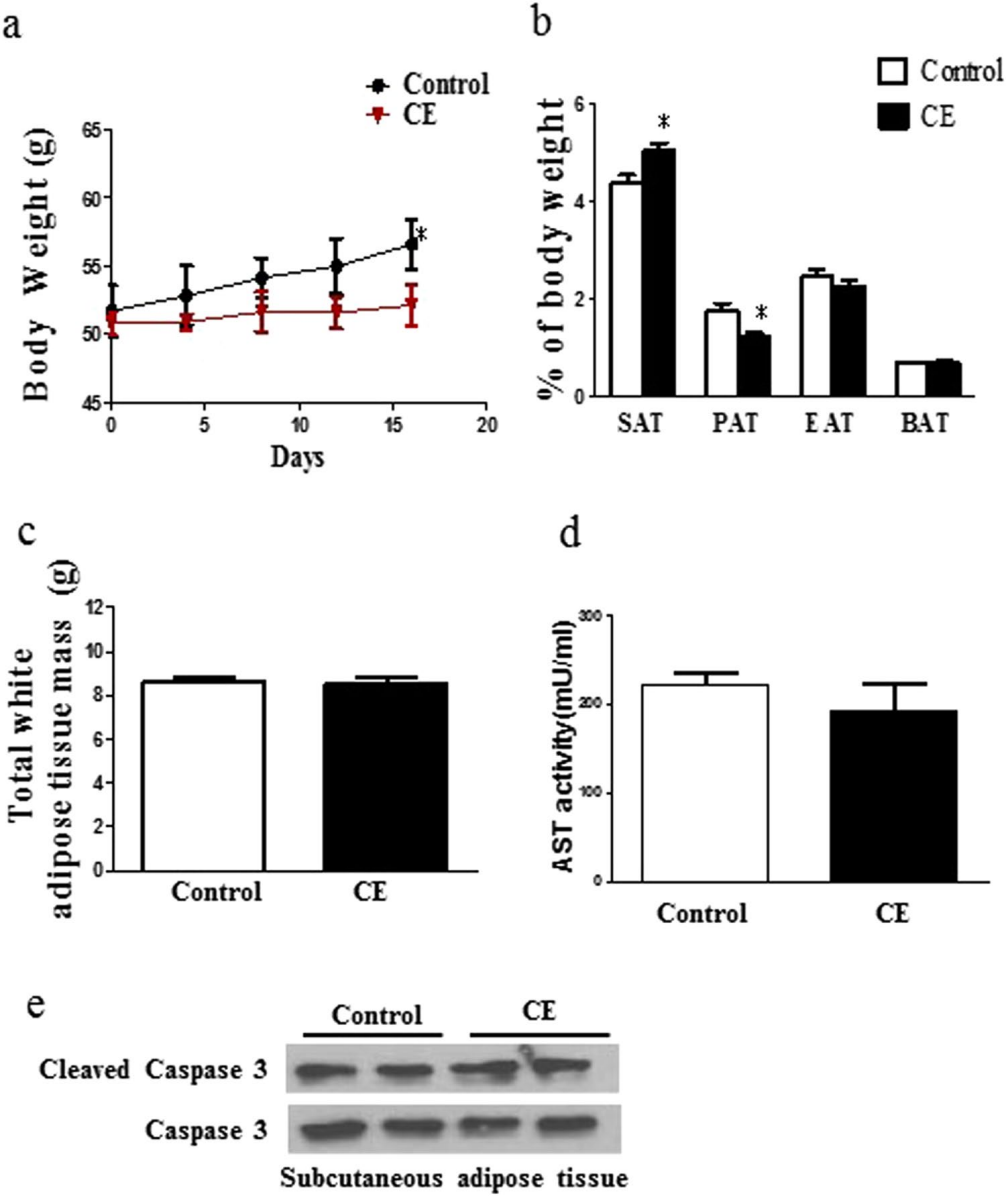
(**a**) Body weight of vehicle control and CE-treated mice. (**b**) Weights of different fat depots, presented in the percentage of body weights. (**c**) Total white adipose tissue mass of the mice. (**d**) Aspartate aminotransferase (AST) levels in the mice. (**e**) Expression of caspase 3 and cleaved caspase 3 in the subcutaneous adipose of the vehicle control and CE-treated mice. CE, cinnamon extract (500 mg/kg body weight). SAT, subcutaneous adipose tissue; PAT, perinephric adipose tissue; EAT, epididymal adipose tissue; BAT, brown adipose tissue. *p < 0.05, n = 3 individual experiments.

**Figure 9:**
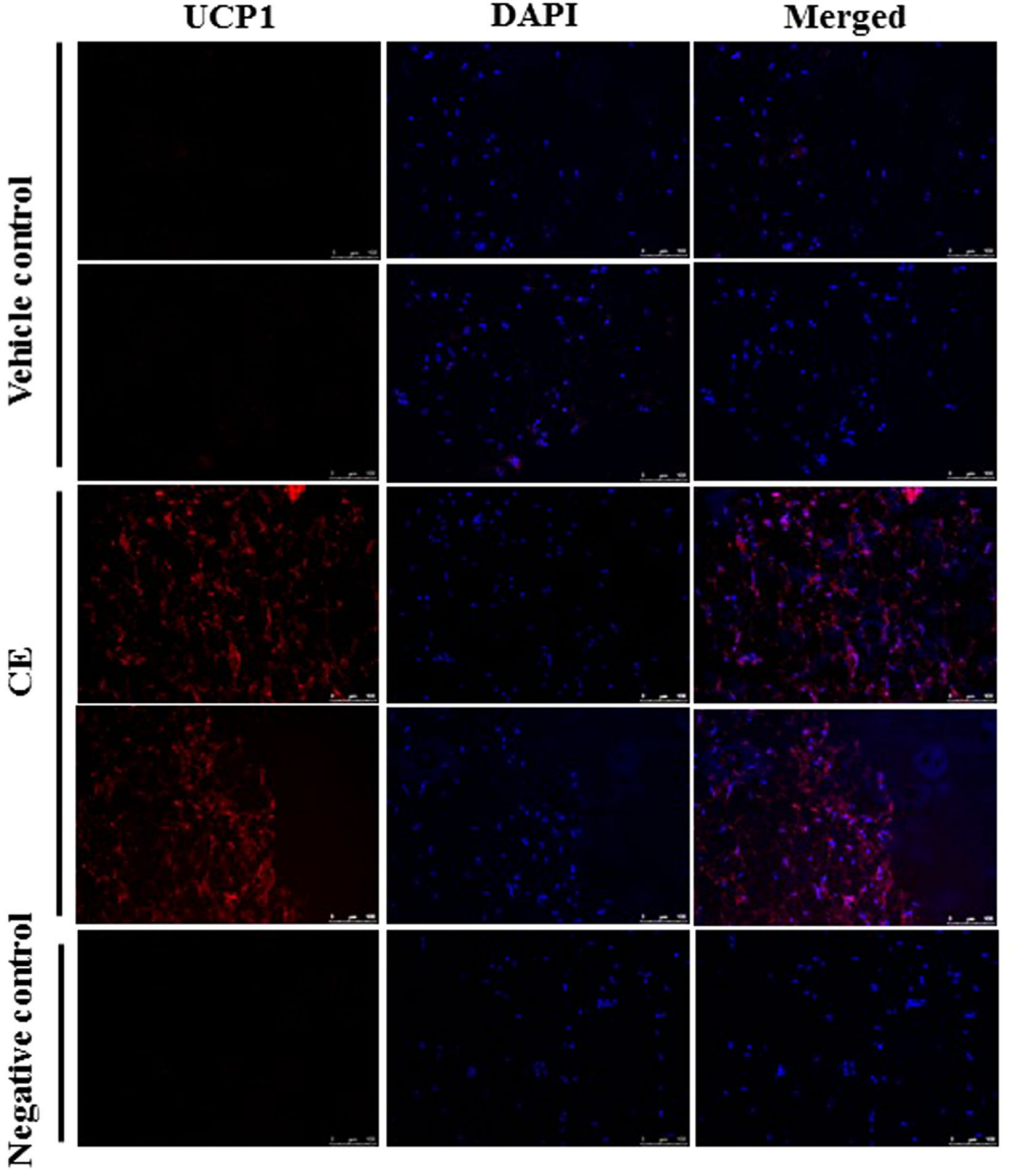
Immunostaining of UCP1 (left panel) and DAPI (4′,6-diamidino-2-phenylindole staining) (middle panel) in the subcutaneous tissues isolated from vehicle control and CE-treated DIO mice. Merged images are shown in the right panel. CE, cinnamon extract (500 mg/kg body weight). UCP1, uncoupling protein 1. Original magnification 20x.

Next, we tried to examine if CE increased Ucp1 expression in subcutaneous adipocytes and reduced body weight *in vivo*. We fed the DIO mice with either vehicle control or CE (500 mg/kg body weight)^26, 27^ for 15 consecutive days. The CE treatment significantly reduced the body weight of the DIO mice (Fig. 8a). The reduction of body weight was unlikely due to changes in organ weight or food intake because the treatment did not have significant effect on organ weight and food intake (data not shown). Although the CE treatment slightly but significantly reduced the mass of perinephric adipose tissue (Fig. 8b) and increased the mass of SAT (Fig. 8B), the total white adipose tissue mass of these mice was not affected (Fig. 8c). The reduction of body weight was unlikely due to detrimental damage of the CE treatment to the mice because the treatment did not affect aspartate aminotransferase (AST) activity (Fig. 8d) or induce apoptosis in the subcutaneous adipose tissue (Fig. 8e and Fig. S7). Interestingly, the CE treatment increased UCP1 expression in the subcutaneous adipose tissue as clearly indicated by the immunostaining (Fig. 9). Therefore, our *in vivo* study suggests that CE treatment increases UCP1 expression in subcutaneous adipose tissue and reduces body weight of the DIO mice.

## Discussion

Upregulation of metabolism and increase heat generation reduce body weight. It is well known that UCP1 is the critical protein for thermogenesis. Our data clearly demonstrated that CE significantly increased the expressions of UCP1 and other brown adipocyte markers in subcutaneous adipocytes and 3T3-L1 adipocytes. The CE treatment also significantly increased mitochondrial protein biogenesis. In the animal study, we found that CE increased UCP1 expression in subcutaneous adipocytes *in vivo* and reduced the body weight of the DIO mice. It is reasonable to postulate that CE increases UCP1 expression and hence the heat production, which leads to a reduction in body weight. Interestingly, the treatment did not significantly affect UCP1 expression in adipocytes isolated from perinephric adipose tissue and epididymal adipose tissue in these mice. Our data also suggest that β3-AR antagonist reduced the CE-enhanced UCP1 expression, suggesting the CE-enhanced UCP1 expression is mediated by β3-AR activity. Our study suggests a natural non-toxic common herbal remedy to reduce obesity.

It is interesting to investigate how CE activates β3-AR. In this study, we used the extract of cinnamon which contains many bioactive compounds. For example, cinnamon contains a lot of derivatives such as cinnamaldehyde, cinnamic acid, cinnamate and numerous polyphenols^12^. Identifying which of these bioactive compounds induce browning by activating β3-AR will be an interesting study. The extract of cinnamon has been shown to have many functions. In animal studies, on top of the antioxidant, anti-inflammatory, antimicrobial and anticancer effects^13^, cinnamon is also found to have beneficial effects in Alzheimer’s disease^12^. However, further investigation is needed to provide more clinical evidence. In other studies, cinnamon lowers glucose and lipid levels in type 2 diabetes patients, it also reduces fasting serum glucose, triglyceride, low density lipoprotein cholesterol and total cholesterol in these patients^28^. However, other human clinical trials with cinnamon resulted in different findings^21^. Nevertheless, cinnamon supplements are sold as preventative or therapeutic supplement for various diseases including type 2 diabetes, metabolic syndrome, insulin resistance, asthma, arthritis, cancers and elevated cholesterol^21^. A study also showed that Cinnulin PF®, a commercial cinnamon extract (Integrity Nutraceutical, USA) improved fasting blood sugar levels, reduced systolic blood pressure and changed body fat composition in human^29^.

Herbal remedies that can induce browning is less studied. Recently, it is reported that berberine induced the development of brown-like adipocytes in inguinal, but not in epididymal adipose depots^30^. Ginsenoside Rb1 promotes browning in 3T3-L1 adipocytes^31^. Here, we reported that cinnamon induced browning in the subcutaneous adipocytes of the obesity mouse models. In agreement with other studies, adipocytes in the other white adipose depots such as perinephric adipose tissue and epididymal adipose tissue cannot be induced to develop brown adipocyte phenotypes. The reason underlying these observations is not known. However, genes in the visceral and subcutaneous adipose tissue of obese subjects are differentially expressed^32^, which may explain their different responses to the same treatment. Indeed, a large accumulation of brite cells during cold exposure can be found in the subcutaneous inguinal adipose tissue, but is rather seldom observed in epididymal or perigonadal adipose tissues^23^.

Interestingly, the origin of the brite cells is controversial. Nowadays, many consider that there are three types of adipocytes—brown, brite and white; while the other consider brite adipocytes are not an independent cell type but they belong to the brown adipocytes. Up to present, there is no evidence suggesting that the functions of brown and brite adipocytes are different. Furthermore, classical brown and brite adipocytes *in vivo* are remarkably alike regarding to both the molecular and morphological markers^7^. Further investigation is needed to find out the origin of the brite cells in our models.

UCP1 is an integral membrane protein unique to brown adipocyte mitochondria, it acts as proton channel to uncouple oxidative phosphorylation by dissipating the proton gradient across the inner mitochondrial membrane. Physiologically, UCP1 is the protein capable of mediating adaptive nonshivering thermogenesis in the cold and it is essential for the recruitment of adaptive adrenergic nonshivering thermogenesis. Therefore, UCP1 enables separation of lipid oxidation from ATP production and allows a higher metabolic rate and conversion of nutritional energy to heat energy. Thermogenic responses in brown fat cells are fully UCP1-dependent. Prdm16 is able to program fibroblast to become brown adipocytes, and subcutaneous implantation of Prdm16 resulted in brown adipocyte-containing fat pads^33^. Prdm16 enhances nuclear receptor-dependent transcription of the brown fat-specific Ucp1 gene^34^. Our study also showed that cinnamon increased Prdm16 expression in 3T3-L1 adipocytes and the subcutaneous adipocytes isolated from db/db mice and DIO mice. Therefore, there is a possibility that CE increases UCP1 expression by increasing Prdm16 expression.

Our study clearly demonstrated that CE treatment induced browning in the subcutaneous adipocytes isolated from obesity mouse models. Since active brown fat is virtually absent or has low thermogenesis activity in obese people, consumption of non-toxic herbal remedies that can induce browning in obese subject should be an attractive strategy to reduce obesity.

## Methods

### Preparation of cinnamon extracts

Cinnamon was purchased from the School of Chinese Medicine, Hong Kong Baptist University. Cinnamon extraction (100 g) was reflux-extracted twice with 70% ethanol (1:10, w/v) for 3 hr each. The extracts were filtered and evaporated under vacuum. The cinnamon extract was analyzed with reversed phase chromatography and Agilent 6540 UHD Accurate Mass Q-TOF LC/MS.

### Animal handling

All animal experimentation was approved and conducted in accordance with the guidelines from Hong Kong Baptist University and was endorsed by the University Human and Animal Subject Committee and the Department of Health, the Government of Hong Kong Special Administration Region. Male mice C57BL/6 (C57) of 5 weeks old and male C57BLKS db/db mice of 5 weeks’ old were purchased form the Chinese University of Hong Kong. C57BL/6 (C57) mice were randomly selected to have either control diet (D12450J Research Diets), or high fat diet (D12762 Research Diets) which was used to induce obesity. Both diet and water were supplied *ad libitum*. Body weight of each mouse was recorded every week. After 3 months of dietary intervention, the diet-induced obesity (DIO) mouse models were used for the experiments.

### Isolation of adipocytes

Isolation of adipocytes from subcutaneous adipose tissues (SAT), perinephric adipose tissue (PAT) and epididymal adipose tissue (EAT) were performed as described elsewhere^35, 36^. Briefly, SAT was dissected from the bilateral superficial subcutaneous white adipose deposits between the skin and muscle fascia just anterior to the lower segment of the hind limbs^35, 36^. PAT is dissected from the adipose capsule of kidney in the mice. EAT is dissected from the fat pad over the epididymis. These fat pads were digested for 1 hr at 37 °C with collagenase in Krebs-Ringer Buffer (12 mM HEPES, 121 mM NaCl, 4.9 mM KCl, 1.2 mM MgSO_4_, and 0.33 mM CaCl_2_) supplemented with 3 mM glucose and 1% fatty acid-free BSA, filtered through nylon mesh. Adipocytes were collected from the upper phase after centrifugation. The isolated adipocytes were counted using a hemocytometer.

### Oil Red-O staining

Lipids are stained by Oil Red O staining. To quantify staining, Oil Red-O was extracted from the cells with isopropanol containing 4% Nonidet P-40, and optical density was then measured at a wavelength of 520 nm.

### 3T3-L1 preadipocyte differentiation

3T3-L1 preadipocytes were induced to differentiate to mature white adipocytes with differentiation inducing medium containing 1 mM dexamethasone, 0.5 mM isobutylmethylxanthine and 1.67 mM insulin in DMEM (Dulbecco’s Modified Eagle’s medium) with 10% FBS for 4 days before switching to DMEM with only 10% FBS and 10 µg/ml insulin for an additional 3 days^37^.

### Western blot

Proteins were prepared as described^36^. Briefly, after boiling the protein for 5 min, the supernatant was separated onto 8% SDS-PAGE and transferred onto nitrocellulose membranes (Bio-Rad). The membranes were then blocked with milk and were incubated with corresponding antibodies (Santa Cruz) overnight at 4 °C. Then, the membranes were washed with Tris-buffered saline (TBS) and TBS-Tween20 (TBST) followed by incubation with corresponding secondary antibodies. The signals were detected by ECL detection system (Amersham Biosciences).

### Real time PCR

Total RNA was isolated using TRIzol reagent (Invitrogen). Reverse transcription was performed with oligo-dT using MMLV reverse transcriptase (Promega, USA) according to the manufacturer’s protocol. Quantitative real time PCR was carried out by monitoring the increase in fluorescence of SYBR green with the ViiA 7 Real Time PCR System (Applied Biosystems, USA). The primer sets were synthesized by Invitrogen. Ucp1, forward: ACTGCCACACCTCCAGTCATT, reverse: CTTTGCCTCACTCAGGATTGG. Cidea, forward: TGCTCTTCTGTATCGCCCAGT, reverse: GCCGTGTTAAGGAATCTGCTG. Prdm16, forward: CAGCACGGTGAAGCCATTC, reverse: GCGTGCATCCGCTTGTG. Cpt1 forward: GCTGGAGGTGGCTTTGGT, reverse: GCTTGGCGGATGTGGTTC. Pparγ forward: GCCCTTTGGTGACTTTATGGA, reverse: GCAGCAGGTTGTCTTGGATG. Pgc1 forward: GCAACATGCTCAAGCCAAAC, reverse: TGCAGTTCCAGAGAGTTCCA. Igf forward: GGACCAGAGACCCTTTGCGGG, reverse: GGCTGCTTTTGTAGGCTTCAGTGG. Dpt forward: GGTGGCTACGGGTACCCATA, reverse: GTCAGAGCCTTCCTTCTTGC. Each sample was amplified in triplicate for quantification. Data were analyzed by relative quantitation using the ∆∆C_t_ method and normalized to glyceraldehyde-3-phosphate dehydrogenase (GAPDH).

### Cryosectioning

Fresh adipose tissues dissected from the mice were cut into slices at 8 µM and fixed with a 10% formaldehyde solution. The formaldehyde was then rinsed before immunostaining^35, 38^. The nuclei were stained with DAPI (4′,6-diamidino-2-phenylindole) (ThermoFisher Scientific). Images were viewed under a fluorescence microscope.

### Transient reporter assay

A ucp1 −6300 bp reporter plasmid was kindly provided by Prof Anne-Marie Cassard-Doulcier (Univ Paris-Sud, Faculté de médecine Paris-Sud, France)^39^. The ucp1 promoter construct from base −2979 to base +76 was ligated into pGL3-basic luciferase reporter vector (Promega). HEK293 cells or 3T3-L1 cells grown in 24-well plates were co-transfected with 0.5 µg of ucp1-luc and 0.02 µg of pRLSV40 encoding Renilla luciferase (rLuc) (Promega). Control cells were co-transfected with 0.5 ug empty pGL3-basic luciferase reporter vector and 0.02 µg pRL-SV40 encoding rLuc. 48 hr-post transfection, cells were treated with cinnamon extract for 24 hr and Dual-Luciferase assay (Promega) was performed. The luciferase readings for each samples were normalized against the rLuc levels^40^.

### cAMP direct immunoassay

Cells were grown in 24-well plates. After treatment, cAMP levels were quantified by cAMP direct immunoassay kit (BioVision) following the manufacturer’s protocol.

### MitoTracker Green staining

Cells were grown in 96-well plates. After treatment, mitochondrial proteins were labeled with MitoTracker® (Molecular Probes) following the manufacturer’s protocol.

### Aspartate aminotransferase activity measurement

The aspartate aminotransferase (AST) activity was measured by the aspartate aminotransferase assay kit (Abcam) following the manufacturer’s protocol.

## Electronic supplementary material

Supplementary Figures
