# Supplementary material for: Cinnamon induces browning in subcutaneous adipocytes
Source: Sci Rep. 2017 May 26;7:2447. doi: 10.1038/s41598-017-02263-5 (PMC5446408; doi:10.1038/s41598-017-02263-5)
Supplement: Supplementary file 1 — Supplementary Figures [file 41598_2017_2263_MOESM1_ESM.pdf]

Cinnamon induces browning in subcutaneous adipocytes

Hiu Yee Kwan[1,2]*, Jiahui Wu[1,2], Tao Su[1,2], Xiao-Juan Chao[1,2], Bin Liu[3], Xiuqiong Fu[1,2], Chi Leung Chan[1,2], Rebecca Hiu Ying Lau[1,2], Anfernee Kai Wing Tse[1,2], Quan Bin Han[1,2], Wang Fun Fong[1,2], Zhi-ling Yu[1,2]*

[1]Centre for Cancer and Inflammation Research, School of Chinese Medicine, Hong Kong Baptist University, Hong Kong, China.

[2]Institute of Integrated Bioinfomedicine & Translational Science, HKBU Shenzhen Research Institute and Continuing Education, Shenzhen, China.

[3]Guangzhou Institute of Cardiovascular Disease, Guangzhou Key Laboratory of Cardiovascular Disease, and the Second Affiliated Hospital, Guangzhou Medical University, Guangzhou, China.

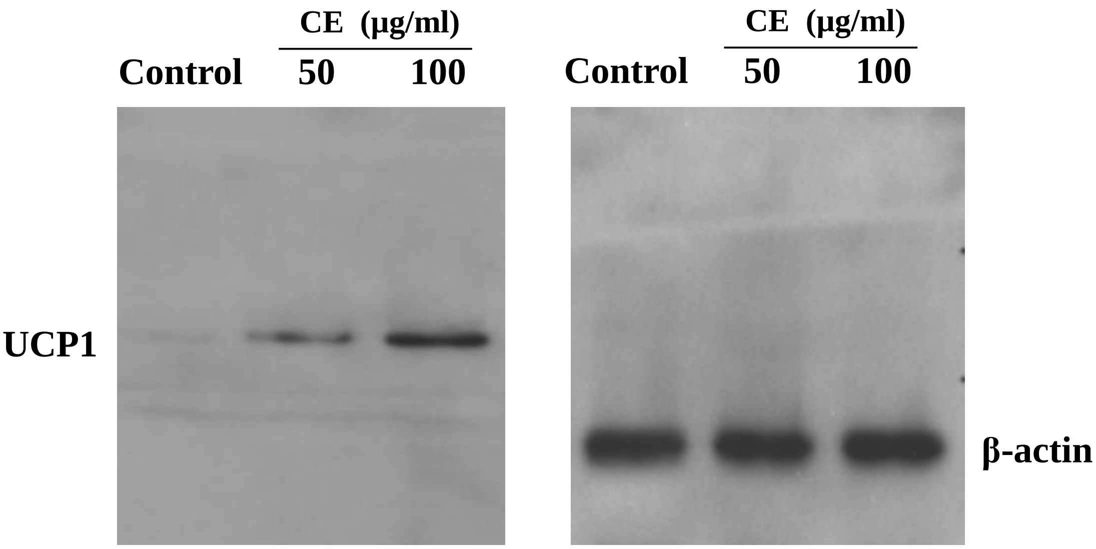

Original Western for Figure 3b
UCP1 protein expression and in vehicle control and cinnamon extract (CE)-treated 3T3-L1 cells.

Supplementary Figure 1

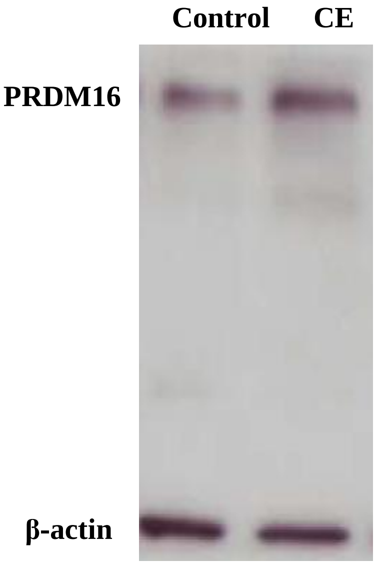

Original Western for Figure 4b.
Protein expression of PRDM16 in vehicle control and CE-treated 3T3-L1 adipocytes

Supplementary Figure 2

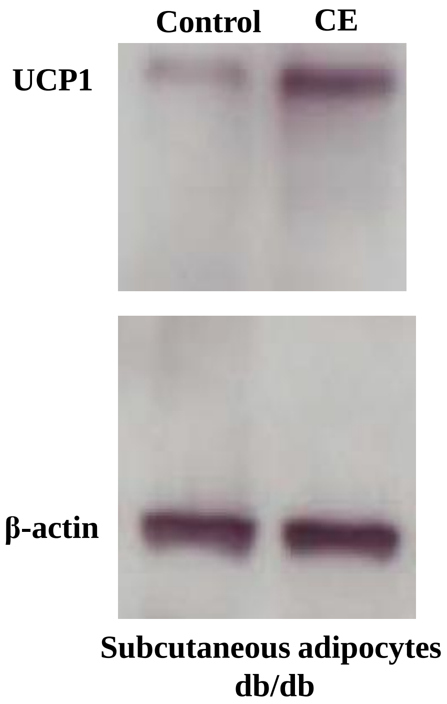

Original Western for Figure 5a
Expressions of UCP1 protein in subcutaneous adipocytes isolated from db/db mice.

Supplementary Figure 3

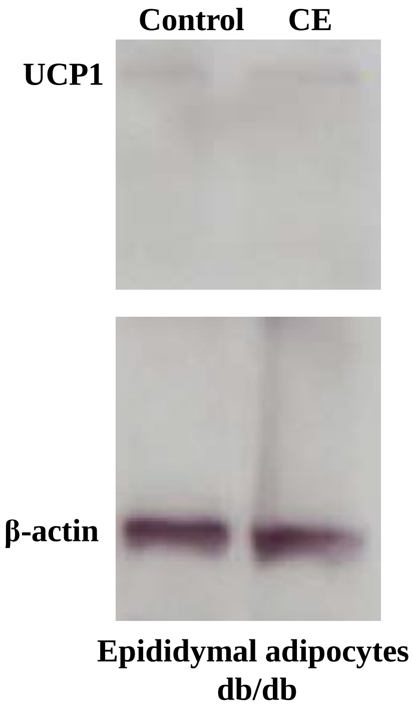

Original Western for Figure 5c

Expressions of UCP1 in epididymal adipocytes isolated from db/db mice.

Supplementary Figure 4

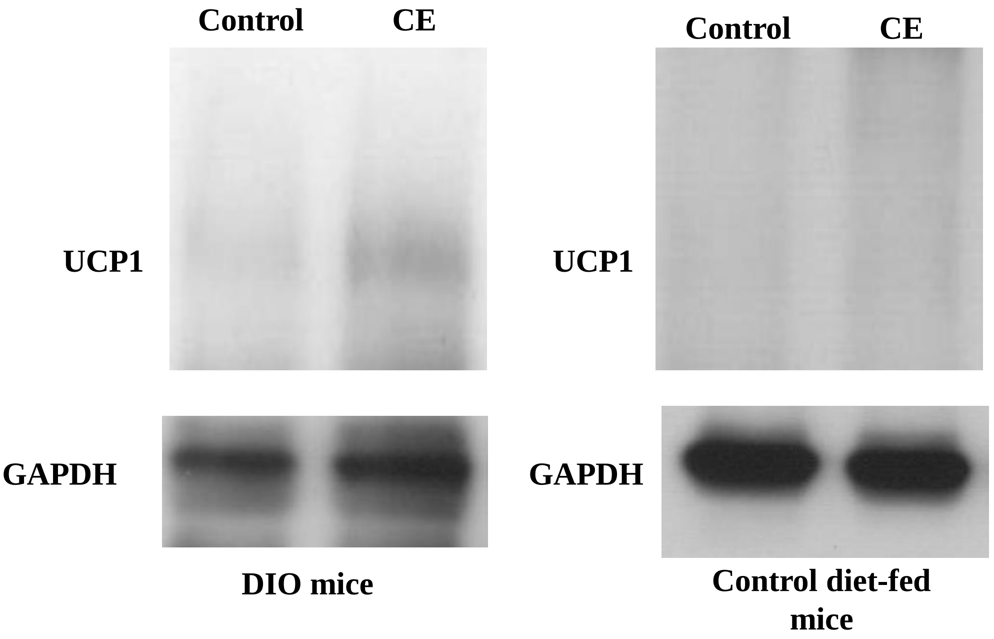

Original Western for Figure 6c
UCP1 protein expression in subcutaneous adipocytes isolated from DIO mice and control diet-fed mice.

Supplementary Figure 5

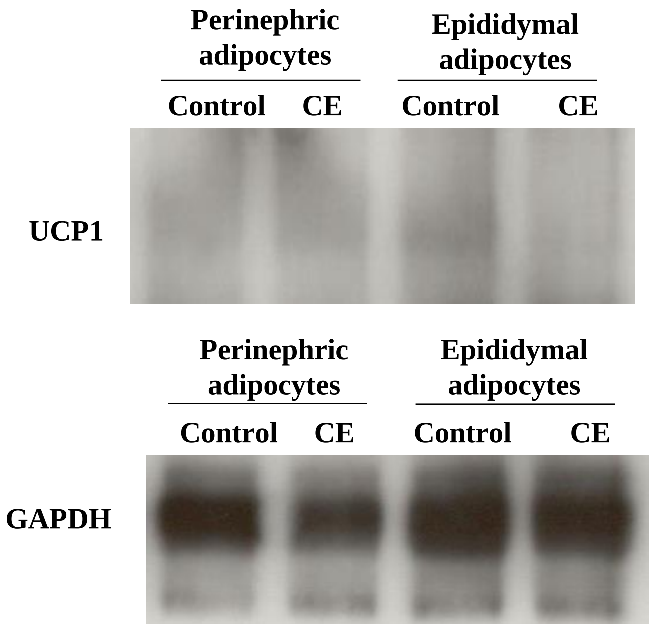

Original Western for Figure 6f
Protein expression of UCP1 in perinephric adipocytes and epididymal adipocytes isolated from DIO mice.

Supplementary Figure 6

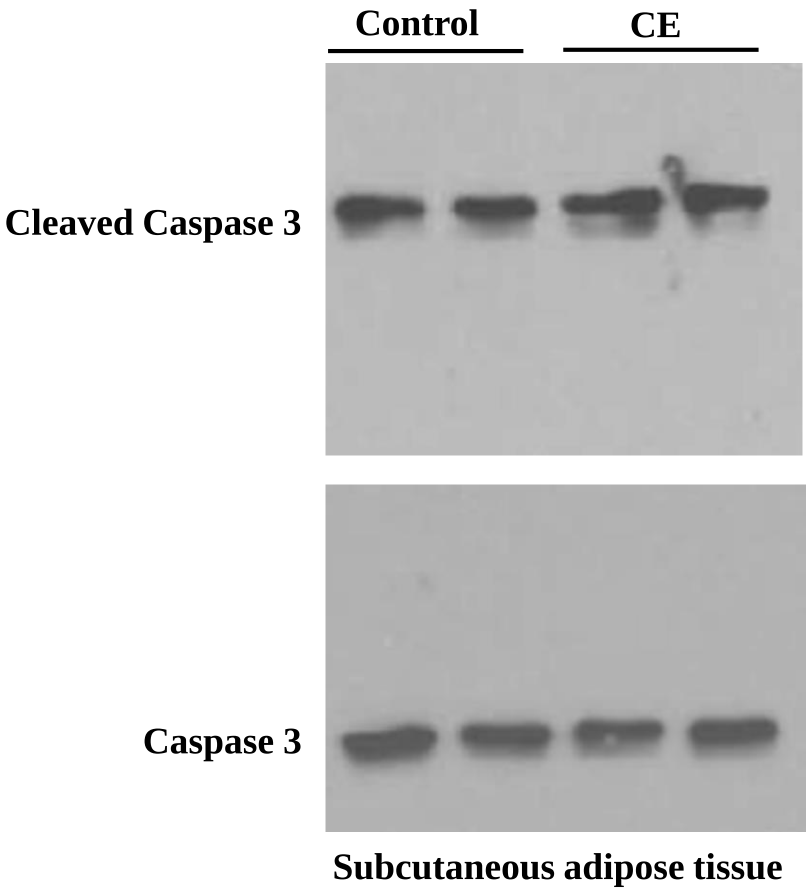

Original Western for Figure 8e
Expression of caspase 3 and cleaved caspase 3 in the subcutaneous adipose in the vehicle control and CE-treated mice.

Supplementary Figure 7
